# Apical Sparing of Longitudinal Strain as a Specific Pattern of Myocardial Fibrosis in Patients with Severe Left Ventricular Hypertrophy: A Comparison between Deformation Imaging and Histological Findings

**DOI:** 10.3390/jcm13206141

**Published:** 2024-10-15

**Authors:** Siarhei Yelenski, Rashad Zayat, Jan Spillner, Srinath Reddy Donuru, Alish Kolashov, Mohammad Amen Khattab, Nima Hatam, Sebastian Kalverkamp

**Affiliations:** 1 Faculty of Medicine, Department of Thoracic Surgery, RWTH University Hospital, RWTH Aachen University, 52074 Aachen, Germany; syelenski@ukaachen.de (S.Y.); jspillner@ukaachen.de (J.S.); skalverkamp@ukaachen.de (S.K.); 2 Heart Centre Trier, Department of Cardiothoracic Surgery, Barmherzige Brueder Hospital, 54292 Trier, Germany; a.kolashov@bbtgruppe.de; 3 Department of Cardiac Surgery, Clinic Nuernberg South, 90471 Nuernberg, Germany; reddyd137@gmail.com; 4 Faculty of Medicine, Department of Cardiac Surgery, RWTH University Hospital, RWTH Aachen University, 52074 Aachen, Germany; mkhattab@ukaachen.de (M.A.K.); nhatam@ukaachen.de (N.H.)

**Keywords:** hypertrophic cardiomyopathy, deformation imaging, echocardiography, histology, myocardial fibrosis, strain analysis, cardiac surgery

## Abstract

**Objectives**: This study aimed to investigate the correlation between apical sparing of longitudinal strain (LS), as measured by speckle-tracking echocardiography (STE), and the histological presence of myocardial fibrosis (MF), in patients with hypertrophic obstructive cardiomyopathy (HOCM). **Methods:** Twenty-seven HOCM patients who underwent elective Morrow procedures +/− aortic valve replacement (AVR) were included. All patients had standard echocardiography, with STE pre- and post-operatively. Intraoperative probes of the interventricular septum were sent for histological analysis. Correlation of different regional LS patterns with the histological findings of MF and with clinical outcome were analyzed. In addition, a logistic regression and ROC analysis were performed. **Results:** All patients underwent the Morrow procedure for HOCM, with 33.3% also undergoing AVR. A total of 74.1% showed evidence of MF in the histological analysis. Patients with MF had significantly lower GLS than patients without MF (−12.7 ± 2.7% vs. −23.0 ± 5.7%, *p* < 0.001). The LS in patients with MF was significantly lower at the basal regions of the LV segments and increased significantly towards the apex as compared to the patients without MF (mean basal-strain %: −10.6 ± 2.6 vs. −17.3 ± 4.6, *p* < 0.001; mean apical strain %: −21.8 ± 4.8 vs. −16.7 ± 5.6, *p* = 0.032). In the logistic regression, only the GLS remained as an independent predictor of MF with an Odds ratio of 1.07 (95%-CI: 1.05–1.09, *p* < 0.001). **Conclusions:** Our study highlights the significant correlation between GLS and MF in HOCM patients. These findings contribute to the growing understanding of MF in HOCM and may inform future approaches to patient management and risk stratification.

## 1. Introduction

Hypertrophic phenotypes of cardiomyopathies are mostly caused by mutations in cardiac sarcomere genes, amounting to 30–60% of all cases [1]. However, they can also occur as a result of cardiac involvement in multi-systemic metabolic illnesses, such as Anderson–Fabry disease (AFD) [2]. Hypertrophic obstructive cardiomyopathy (HOCM) carries a 0.9% annual risk of sudden cardiac death (SCD) and is the leading cause of SCD among young adults [1,3].

HOCM is characterized by left ventricular (LV) hypertrophy of at least 15 mm in any segment that cannot be attributed to aberrant loading conditions [4]. Approximately 70% of patients with HOCM experience dynamic blockage inside the left ventricle either at rest or upon stimulation [4,5]. In obstructive hypertrophic cardiomyopathy (HOCM), the primary gradient is predominantly situated in the left ventricular outflow tract (LVOT) at the junction of the hypertrophied interventricular septum and the systolic anterior motion (SAM) of the mitral valve [6,7].

Heterogeneous myocardial hypertrophy and cardiomyocyte derangement are recognized as significant histopathological features of HOCM, in addition to myocardial fibrosis (MF) [8], which is a progressive, irreversible process that leads to myocardial systolic and diastolic dysfunction, as well as electrical inhomogeneity [9].

Clinically, myocardial biopsy is the best way to identify MF. Myocardial biopsy is rarely utilized to detect MF due to its invasiveness and limited sampling sites [10]. However, cardiac magnetic resonance late gadolinium enhancement (CMR LGE) can identify myocardial fibrosis non-invasively and offer HOCM patients with prognostic information. Regrettably, the clinical use of CMR LGE remains limited due to constraints related to the exorbitant expenses involved and the adverse reactions caused by contrast agents [11].

In recent years, advances in echocardiography technology have enabled the assessment of myocardial deformation imaging using speckle-tracking echocardiography (STE), which has shown promising results in identifying early stages of LV dysfunction in HOCM [12,13,14]. Deformation echocardiography is a valuable non-invasive tool that enables the detection of subclinical changes in myocardial function, even in the absence of LV systolic dysfunction [15,16,17]. A pattern of apical sparing of longitudinal strain (LS) has been previously described in storage diseases such as cardiac amyloidosis (CA) [18,19].

Standard echocardiography of patients with HOCM and CA reveals similarities in LV chamber size and wall thickness, but advanced imaging identifies variations in LV function [20,21]. While STE derived global longitudinal strain (GLS) effectively detects subclinical LV failure in patients with hypertrophic morphologies, it is limited by its load dependency. Recently, a novel non-invasive analysis of pressure-strain loop-derived myocardial work (MW) has been suggested as an approach for evaluating myocardial function, addressing the limitations of GLS [22].

Recent studies demonstrated that the non-invasive MW analysis is a valid contemporary method for examining cardiac function in individuals with hypertrophic phenocopies. The global myocardial work index (GWI) and global constructive myocardial work (GCW) were able to differentiate patients with HOCM from CA patients [23,24].

This study aimed to investigate the correlation between apical sparing of LS, as measured by STE, and the presence of MF, as assessed by histological examination, in patients with HOCM. We intended to improve our understanding of the clinical importance of this discovery and its potential usefulness in risk evaluation and surgical decision-making.

## 2. Materials and Methods

### 2.1. Patient Population

**Figure 1:**
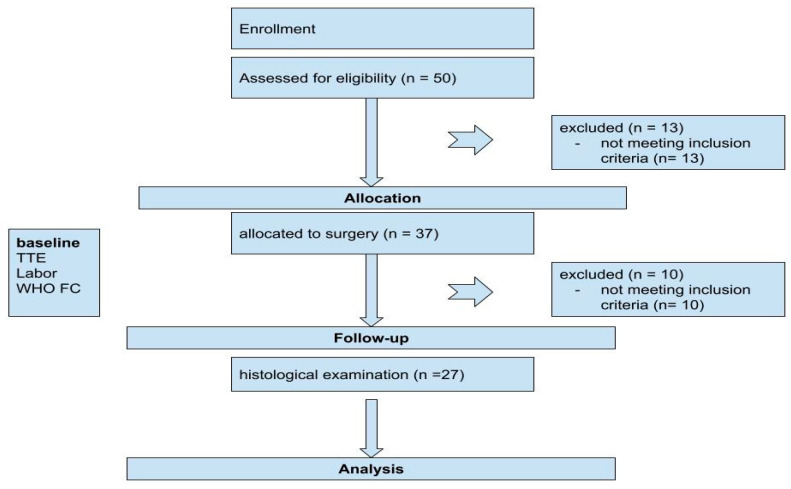
Study design flow chart. TTE: transthoracic echocardiography. WHO FC: the World Health Organization functional class.

This was a retrospective analysis of prospectively collected data from all consecutive patients who were referred to our echocardiography laboratory with a diagnosis of HOCM between 2016 and 2023. Inclusion criteria included patients accepted for elective surgery for the Morrow procedure with a diagnosis of HOCM based on 2D echocardiographic evidence of wall thickness ≥ 15 mm and significant LVOT obstruction (resting or maximum provoked LVOT gradient of ≥50 mmHg), clinical angina, dyspnea (NYHA functional class III-IV), and syncope [5]. Exclusion criteria were previous myocardial infarction, structural heart disease, and the absence of a sufficient quality of a transthoracic echocardiogram (TTE) or histologic sample. A total of 50 patients were screened and 27 met the inclusion criteria and were enrolled in the study (Figure 1). Twenty-three patients were excluded due to the following: previous myocardial infarction (n = 12), insufficient quality of TTE or absence of histologic sample (n = 10), or structural heart disease (n = 1). Ethical approval was obtained from the Research Ethics Committee of RWTH University Aachen, Germany (EK 151/09). Informed consent was obtained from all patients.

### 2.2. Echocardiography

A TTE is included in our regular patient evaluations. All patients had a standardized TTE before and after surgery. A TTE was conducted the day prior to the surgery and on the 7th postoperative day (POD). Patients were scanned in the left lateral decubitus position using typical 2D pictures capturing three cardiac cycles triggered to the QRS complex and preserved in cine-loop digital format for further analysis. The comprehensive TTE was performed included a complete standard M-mode and 2D echocardiographic exams including tissue-Doppler imaging (TDI) and STE according to the American Society of Echocardiography and the European Association of Cardiovascular Imaging [25]. It also included various measurements such as the ejection fraction (EF) using the Simpson biplane method from apical two- and four-chamber windows, left atrial volume index using a biplane formula, end-diastolic interventricular septal position (ITS), relative wall thickness (RWT), and LV inner dimension in the diastole. Diastolic parameters, such as peak early (E) and late (A) diastolic mitral inflow velocity and its ratio (E/A), deceleration time (DT), and average of the medial and lateral mitral annular diastolic velocities (e′), were also assessed. All echocardiography studies were performed using the Vivid E9 (GE Vingmed Ultrasound AS, Horten, Norway) and the measurements were determined using the EchoPAC version BT 113 (GE Vingmed Ultrasound AS, Horten, Norway). For deformation analysis, automated software (EchoPAC^®^, Version 202; GE Medical Systems, Horten, Norway) was used offline to generate a “bull’s eye” plot showing segmental LS values. A global LS value was obtained by averaging the strain values from all segments, and an apex-to-base gradient in regional LS was calculated based on absolute strain values. Additionally, a relative apical LS was calculated by dividing the average apical LS by the sum of the average basal LS and the average mid LS. TTE studies and analyses were performed by experienced and certificated physicians.

### 2.3. Histology

After surgery, surgical specimens of interventricular septum (IVS) were collected for histological examination. The samples were fixed in 10% formalin and embedded in paraffin. Sections were stained with Masson’s trichrome stain to evaluate the extent of fibrosis. Congo red staining was used to detect whether amyloid fibril deposits were evident or not. The percentage of fibrosis was determined by a pathologist who was blinded to the patients’ echocardiographic and clinical data. Inter-observer and intra-observer variability were assessed by repeating the measurements in 10 randomly selected patients by two independent observers or one observer, respectively. The mean percentage of fibrosis was used for further analysis.

### 2.4. Outcomes

The primary outcome of this study was to analyze the correlation between the calculated LS from different regions of the LV and the apical sparing visualized in the bull’s-eye plot of STE in patients with LV hypertrophy secondary to HOCM according to the histological findings. Secondary outcomes included assessing the clinical significance of this finding and its potential use in risk stratification and surgical decision-making.

### 2.5. Statistics

All statistical analyses were performed utilizing R statistics (Version 3.6, Vienna, Austria) and the Jamovi project (Version 2.3.2, https://www.jamovi.org, accessed on 20 March 2024). Regarding categorical variables, absolute values and percentages were employed. To evaluate the normality of the distribution of continuous variables, the Kolmogorov–Smirnov test was applied. We employed the median and interquartile range (IQR) to represent non-normally distributed variables, while the mean and standard deviation were utilized for normally distributed data. For continuous variables, comparisons of non-normally distributed data were conducted using the Mann–Whitney U-test. To compare groups based on normally distributed non-repeated continuous variables, two-tailed Student’s *t*-tests were applied. The exact test of Fisher was applied to categorical variables. Spearman’s correlation was performed to evaluate the correlation between echocardiographic parameters and the incidence of MF.

To identify echocardiographic parameters as predictors of MF in HOCM patients, generalized linear logistic regression was performed. The entry criteria for the multivariate analysis were a *p* value < 0.05 in the univariate analysis. The receiver operator characteristic (ROC) curve and area under the curve (AUC) from each independent predictor were examined. All statistical comparisons were two-sided, with *p* values < 0.05 considered significant. Inter-observer and intra-observer variability were analyzed using intra-class correlation coefficients.

## 3. Results

All patients with amyloid fibril depositions were excluded. The study included 27 patients with a mean age of 66.4 ± 12.9 years, of which 55.6% were female. All patients underwent the Morrow procedure for HOCM, with 33.3% also undergoing aortic valve replacement for structural valve disease and 22.2% undergoing coronary artery bypass grafting for concomitant coronary artery disease (Table 1). Table 1 summarizes the baseline characteristics of the study population.

The mean length of stay in the clinic after surgery was 16.3 ± 10.2 days, with a mean ICU stay of 6.4 ± 10.5 days. Among the 27 patients, 20 (74.1%) had fibrosis confirmed in histological samples, while 7 (25.9%) did not show any fibrosis. Table 2 presents the postoperative course and complications.

### 3.1. Echocardiographic Results

The echocardiographic results revealed that the mean EF was 64.07 ± 6.7%, but the mean GLS was slightly reduced compared to normal at −15.4 ± 5.8% [26]. The study patients showed signs of diastolic dysfunction, as evidenced by a mean E/A ratio of 1.07 ± 0.54 and a mean DT of 250.2 ± 96.3 ms. Additionally, the patients had severely enlarged left atria, with a mean left atrial volume index (LAVI) of 49.6 ± 16.7 mL/m^2^, indicating significant impairments in cardiac diastolic function. Table 3 demonstrates all measured TTE values. The patients had concentric left ventricular hypertrophy, as indicated by a mean left ventricular mass index (LVMI) of 198.7 ± 58.5 g/m^2^ and a mean RWT of 1.2 ± 0.7 cm. The mean pressure gradient (MPG) and V_max_ of LVOT were both within the normal range at 8.6 ± 12.6 mmHg and 1.75 ± 0.9 m/s, respectively. However, the MPG over the aortic valve (AV) was 41.2 ± 27.5 mmHg, and the V_max_ over the AV was 4.05 ± 1.6 m/s, suggesting significant aortic stenosis. Additionally, the mean right ventricular systolic pressure (RVSP) was 26.8 ± 5.9 mmHg, indicating mild pulmonary hypertension.

### 3.2. Two-Dimensional Speckle-Tracking Analysis

**Figure 2:**
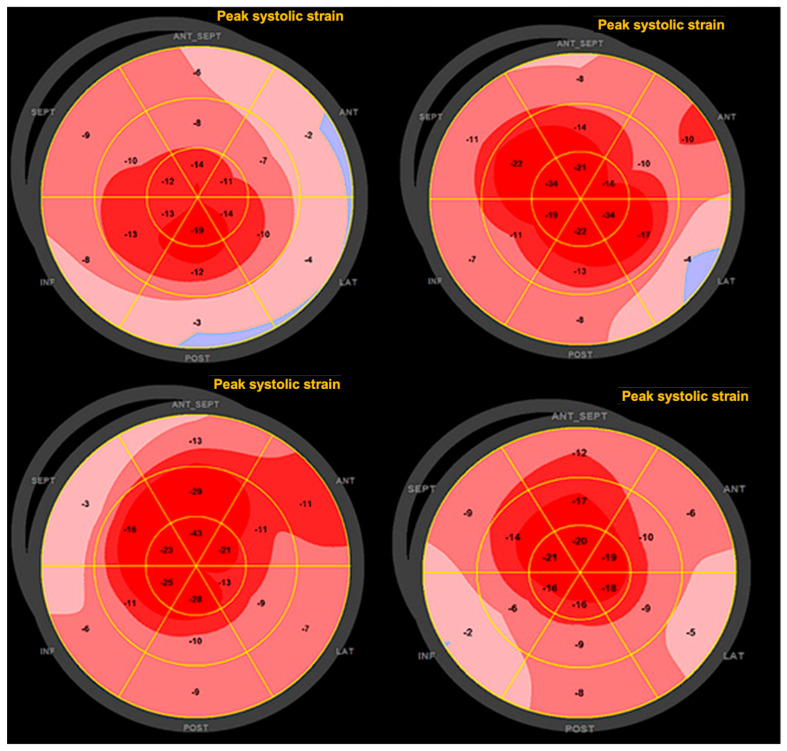
Representative two-dimensional speckle-tracking longitudinal strain patterns (‘bull’s eye plots’). In patients with hypertrophic cardiomyopathy or cardiac amyloidosis, longitudinal strain bull’s eye mapping shows this relative apical sparing. Patients can present with normal EF and have a bull’s eye plot with a normal or slightly reduced average longitudinal strain, a normal longitudinal strain value at the LV apex (bright red), and a markedly reduced strain at all basal segments (pink or blue).

Table 4 presents the STE analysis parameters of the study population and Figure 2 provides a representative STE analysis with the bull’s eye plots. The mean basal strain was found to be −9.5 ± 11.7%. Similarly, we observed a mean mid-strain of −14.43 ± 3.29%. The mean apical strain was found to be −20.45 ± 5.41%. In terms of relative apical strain, we found a mean value of 0.91 ± 0.30. Finally, we examined the base-to-apex gradient of LS, which had a mean value of −12.69 ± 14.25 mmHg.

Overall, our findings demonstrate significant differences in LS parameters across the left ventricle in HOCM patients relative to normal values, with notable variations in the strain values at different segments of the ventricle.

### 3.3. Histological Findings

The histological analysis of the study population revealed a significant presence of severe mid-wall fibrosis in the myocardium. Among the 27 patients included in the study, 20 (74.1%) showed evidence of fibrosis, while 7 (25.9%) did not exhibit any fibrotic changes.

The finding of severe mid-wall fibrosis underscores its potential impact on cardiac function and overall prognosis in patients with this condition. The presence of fibrosis can lead to increased myocardial stiffness and reduced contractility, contributing to the characteristic features of left ventricular hypertrophy and diastolic dysfunction observed in the study population.

#### Comparison between Patients with Identified Histological MF and Patients without MF

Patients with histological MF had significantly lower GLS than patients without MF (−12.7 ± 2.7% vs. −23.0 ± 5.7%, *p* < 0.001). The LS in patients with confirmed MF was significantly lower at the basal regions of the LV and increased significantly towards the apex as compared to the patients without confirmed MF (mean basal-strain %: −10.6 ± 2.6 vs. −17.3 ± 4.6, *p* < 0.001; mean apical strain %: −21.8 ± 4.8 vs. −16.7 ± 5.6, *p* = 0.032) (Table 5). The relative apical strain was significantly higher in MF group (1.0 ± 0.3 vs. 0.6 ± 0.1, *p* = 0.003). Both the maximal pressure over the aortic valve and the maximum velocity were significantly higher in the patients with confirmed histological MF (Table 5).

### 3.4. Correlation Analysis

**Figure 3:**
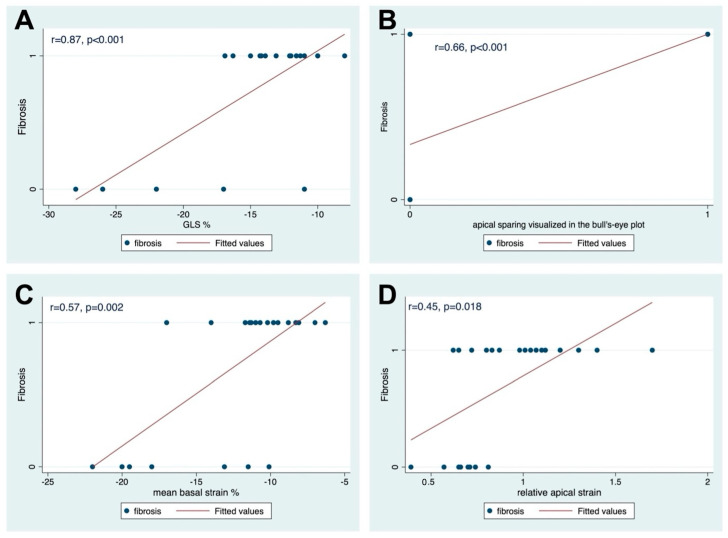
Scatter plots of the correlation matrix. (**A**) the correlation between global longitudinal strain and confirmed histological fibrosis; (**B**) the correlation between apical sparing visualized in the bull’s-eye plot and histological fibrosis; (**C**) the correlation between mean basal-strain % confirmed histological fibrosis; and (**D**) the correlation between relative apical strain and confirmed histological fibrosis.

Figure 3 summarizes the correlation analysis results. In the correlation analysis we found that the GLS correlated very well with the confirmed histological finding of MF (*r* = 0.87, *p* < 0.001). Mean basal-strain % correlated as well with the incidence of MF (*r* = 0.57, *p* = 0.002). Relative apical strain showed a weak correlation with the incidence of MF (*r* = 0.45, *p* = 0.018). The apical sparing visualized in the bull’s-eye plot in speckle-tracking echocardiography correlated well with the confirmed histological MF (*r* = 0.66, *p* < 0.001).

After entering all variables with a *p* < 0.05 from the univariate analyses into a generalized linear logistic regression model (Table 6), only the GLS remained as an independent predictor of MF with an Odds ratio of 1.07 (95% confidence interval: 1.05–1.09, *p* < 0.001).

**Figure 4:**
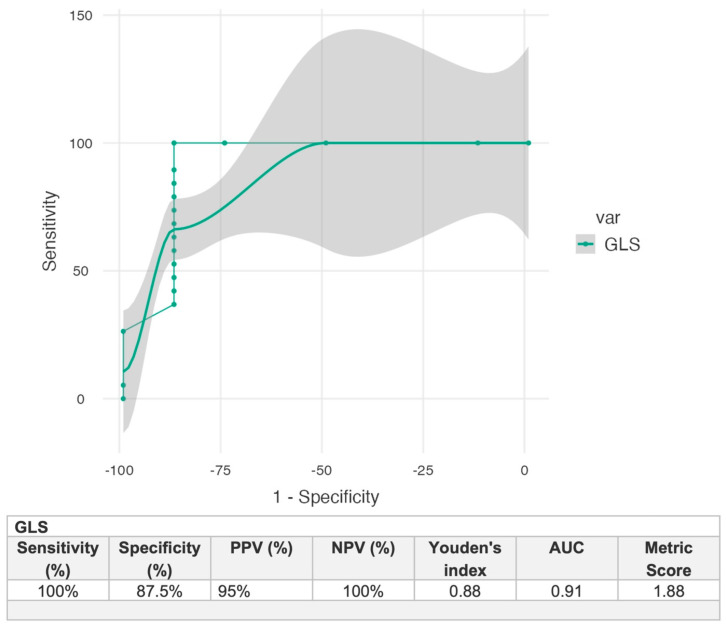
The ROC analysis of the independent predictor of the incidence of histological myocardial fibrosis.

The ROC analysis (Figure 4) demonstrated high accuracy with an area under the curve of (0.91) for GLS, indicating excellent discriminatory power. GLS correctly classified 95% of cases and showed perfect sensitivity (100%) and a specificity of (87%) in identifying patients with MF.

## 4. Discussion

This study investigated the potential use of STE strain parameters provided by echocardiography for early diagnosis and evaluation of MF in individuals with HOCM. The main finding are as follows: Firstly, patients with HOCM and confirmed histological MF showed in comparison to patients without confirmed MF a different LS distribution in the LV with significantly lower LS values at the basal regions of the LV with a gradual increase of LS values towards the apex. Furthermore, GLS was the only independent predictor to accurately forecast substantial MF and had a sensitivity (100%) and a specificity of (87%).

Several investigators have conducted STE over the past decade to investigate the correlation between strain parameters and MF in HOCM patients [27,28]. MF assessment can improve HOCM risk classification and prognosis. HOCM patients may benefit from strain imaging to detect impaired heart function and differentiate between non-MF and MF segments [29,30].

The investigation of the echocardiographic findings in HOCM patients provided valuable insights into the pathophysiology and clinical implications of this complex cardiac condition. A critical aspect of our study was the comparison between echocardiographic parameters and histological findings, particularly regarding MF.

The evaluation of left atrial (LA) structure and function is crucial for assessing LV filling in several cardiovascular disorders, including hypertension, heart failure, valvular heart disease, and cardiomyopathies [31]. STE is employed to assess LA function, with LA strain, represented by the LA strain curve featuring two positive peaks indicative of LA-reservoir and LA-pump function, acting as a sensitive marker of LV filling pressure, surpassing LA volume, and facilitating the early identification of preclinical LV dysfunction and remodeling [32,33]. Monte et al. [31] demonstrated in their study that LA function evaluated by STE is markedly compromised in patients with coronary artery disease compared to those with HOCM and healthy controls. These findings underscore the possible auxiliary function of STE in the early identification and management of the disease.

The prognostic value of MF in HOCM patients has been well-established in the literature [14,34,35,36]. The presence and extent of significant MF are associated with an increased risk of adverse events, including arrhythmias and HF exacerbations [14,37]. We found that GLS correlated very well with the confirmed histological incidence of MF. This correlation underscores the clinical relevance of assessing regional MF, as it indicates a more advanced stage of HOCM. Our analysis of LS parameters across the LV revealed significant differences in strain distribution, indicating regional variations in myocardial function. These findings, coupled with the histological analysis showing a significant presence of severe mid-wall fibrosis in the myocardium, underscore the complex interplay between structural abnormalities and MF in HOCM. Our findings are in accordance with Popović et al. [38], who found that in HOCM patients with a preserved LV ejection fraction, that there is a correlation between MF and segmental strains using CMR LGE and 2D-STE. The results indicated that the mean LS was correlated with the number of myocardial segments suffering from MF (r = 0.47, *p* < 0.005) and the extent of the MF (r = 0.46, *p* < 0.005) [38].

The observed correlation between GLS and MF, along with GLS being identified as an independent predictor of MF in logistic regression analysis, underscores the clinical relevance of GLS in assessing MF in HOCM. Specifically, our findings demonstrate that reduced GLS values are strongly associated with the presence of MF in HOCM patients. In accordance to our findings, Hu et al. [39] found that GLS could predict significant MF at a cutoff value of −16.5% with a sensitivity of 80.9% and specificity of 76.5%, based on a study with 61 patients [39]. Importantly, GLS may offer valuable prognostic information beyond traditional echocardiographic parameters, as impaired GLS is associated with adverse outcomes in HOCM [40,41]. Furthermore, our findings suggest that segmental LS parameters, such as relative apical strain, may also provide additional insights into the presence and distribution of MF. The correlation between relative apical strain and MF highlights the potential utility of segmental strain analysis in MF assessment, warranting further investigation in larger cohorts.

CMR imaging is now the primary method for differentiation of hypertrophic phenocopies. Its combined CMR sequence evaluation allows tissue characterization. CMR confirms morphological traits more accurately and reproducibly than an echocardiogram [42]. The late gadolinium enhancement (LGE) on CMR can help define the etiology and prognostic classification of numerous hypertrophic phenocopies [42].

Multiparametric tissue characterization makes CMR useful for hypertrophic phenocopies differential diagnosis. Over the past decade, T1 and T2 mapping have permitted quantitative and comprehensive cardiac tissue characterization [42]. Mapping improves diagnostic accuracy and quantifies medication management, especially in infiltrative cardiomyopathies [42]. While CMR is considered the “gold standard” for detecting myocardial fibrosis, it has notable limitations, including the risk of nephrogenic systemic fibrosis (NSF) in patients with severely impaired renal function and potential interference with cardiac implantable electronic devices (CIEDs) [43,44,45]. Additionally, practical challenges such as claustrophobia, high costs, and limited access to specialized centers further restrict its routine clinical use. Given these challenges, STE has proven to be a safer, non-invasive, and more accessible alternative for detecting early myocardial dysfunction, particularly in HOCM patients. For at-risk relatives, initial screening with electrocardiography (ECG) and echocardiography, ideally reviewed by an HOCM specialist, could identify early signs such as left ventricular hypertrophy or systolic outflow obstruction [46]. Early identification of myocardial fibrosis in these individuals can facilitate timely intervention and further genetic or cascade screening in other family members, without exposing patients to the potential adverse effects of gadolinium.

In this context, STE could be integrated into routine screening protocols for at-risk individuals, providing a safer, repeatable, and cost-effective alternative to CMR with LGE. This non-invasive tool could thereby enhance early diagnosis and management strategies, ultimately improving clinical outcomes in both HOCM patients and their high-risk relatives.

The assessment of MF, particularly using late gadolinium enhancement (LGE) on cardiac magnetic resonance imaging (MRI), has emerged as a valuable tool for risk stratification in HOCM patients [34,47,48,49]. A recent meta-analysis has confirmed the prognostic value of LGE, revealing an increased risk for adverse clinical outcomes in patients with significant fibrotic burden [50]. Over the past decade, several studies utilizing STE have explored the association between strain parameters and MF in HOCM patients [36,39,41,47]. These investigations consistently found that segments with LGE, indicative of MF, exhibited significantly reduced segmental LS compared to segments without LGE, highlighting the impact of fibrosis on myocardial deformation. It is important to note that increased LV wall thickness, a hallmark feature of HOCM, can also be observed in other cardiac and systemic diseases. The pathophysiological pattern of increased LV wall thickness ranges from HOCM to conditions such as hypertensive heart disease, cardiac amyloidosis, Anderson–Fabry disease, and physiological hypertrophic athlete’s heart [15]. The presence of MF in patients with afterload-generated hypertrophy is a crucial hallmark of disease severity [51,52]. A notable example of such LV hypertrophy is seen in aortic stenosis, one of the most common valvular diseases in the western world [53,54]. Aortic stenosis is characterized not only by progressive valve obstruction but also by LV remodeling in response to pressure overload [55,56]. This hypertrophic response initially maintains myocardial performance, but over time, it can lead to decompensation and HF, necessitating consideration of aortic valve replacement (AVR). While current guidelines for AVR focus on the severity of valve involvement and the presence of HF symptoms, the role of MF and its impact on long-term survival is increasingly recognized [57,58,59]. Existing research highlights the prognostic significance of MF and suggests its potential as an early marker of LV decompensation, especially in asymptomatic patients [60]. Nonetheless, the diagnosis of MF in these patients remains challenging, particularly using echocardiography, which is more accessible but less sensitive than MRI. Our research addresses this gap by utilizing deformation analysis to diagnose MF, offering a non-invasive and potentially earlier marker of LV decompensation. However, further investigations are warranted to validate these findings and elucidate the role of echocardiography in diagnosing and monitoring MF in patients with HOCM and other conditions characterized by LV hypertrophy.

### Limitations

In discussing our findings, it is important to acknowledge certain limitations that may impact the interpretation and generalization of our results:

1. Sample size and selection bias: Our study included a relatively small sample size, which may limit the generalizability of our findings to broader populations of HOCM patients. Additionally, the patients included in our study were recruited from a single center, which may introduce selection bias and limit the extrapolation of our results to other settings or populations.
2. Histological assessment: While histological analysis is considered the gold standard for assessing MF, our study relied on a single biopsy or imaging modality to evaluate fibrotic burden. This approach may not capture the full extent or distribution of fibrosis within the myocardium, potentially underestimating its prevalence or significance.
3. Echocardiographic techniques: Although echocardiography is a widely used and valuable tool for assessing cardiac structure and function, it has inherent limitations, such as variability in image quality and operator dependency.
4. Exclusion criteria: Our study may have excluded certain subgroups of HOCM patients, such as those with comorbidities or contraindications to invasive procedures like endomyocardial biopsy. This could potentially limit the generalizability of our findings to the broader HOCM population.

## 5. Conclusions

Our study contributes to the growing body of evidence highlighting the importance of non-invasive assessment, particularly using echocardiography and deformation analysis, in diagnosing MF and monitoring disease progression. By improving our ability to detect MF earlier in the disease process, we can potentially optimize patient care and outcomes in conditions such as HOCM and aortic stenosis. Furthermore, our study highlights the significant correlation between GLS and histologically proven MF in HOCM patients. GLS was an independent predictor of MF in HOCM patients, which underscores the clinical relevance of using 2D-STE in assessing regional fibrosis as a marker of disease severity and adverse outcomes.

## Figures and Tables

**Table 1:** Patients’ characteristics and preoperative data.

Characteristics	
Age (years)	66.37 ± 12.89
male n (%)	12 (44.4%)
BMI kg/m^2^	29.53 ± 5.55
Aortic Valve Replacement n (%)	9 (33.3%)
Coronary Artery Bypass Grafting n (%)	6 (22.2%)
Atrial fibrillation n (%)	6 (22.2%)
Chronic Obstructive Pulmonary Disease n (%)	3 (11.1%)
Diabetes mellitus n (%)	7 (25.9%)
Hypertension n (%)	24 (88.9%)
Hyperlipidemia	22 (81.5%)
NYHA II	21 (77.8%)
NYHA III	6 (22.2%)
WBC (10^9^/L)	9.09 ± 3.23
Hb (g/dL)	11.2 ± 1.91
Platelets (10^9^/L)	314 ± 103
LDH U/L	332 ± 123
Creatinine (mg/dL)	0.969 ± 0.306
GFR (mL/min)	73.4 ± 24.1
Potassium (mmol/L)	4.38 ± 0.586

BMI: body mass index, kg/m^2^; NYHA: New York Heart Association; WBC: white blood cell count; Hb: hemoglobin; LDH: lactate dehydrogenase; and GFR: glomerular filtration rate.

**Table 2:** Postoperative outcomes.

Length of stay (days)	16.31 ± 10.18
ICU stay (days)	6.41 ± 10.53
Bleeding/Re-thoracotomy (%)	4 (14.8%)
Stroke (%)	2 (7.4%)
Cardiogenic shock n (%)	2 (7.4%)
CVVH n (%)	2 (7.4%)
Pneumonia n (%)	8 (29.6%)
Delirium n (%)	7 (25.9%)
Pacemaker n (%)	2 (7.4%)

CVVH: Continuous veno-venous hemofiltration. ICU: intensive care unit.

**Table 3:** Echocardiographic characteristics.

Parameters	
EF, %	64.07 ± 6.72
GLS, %	−15.39 ± 5.83
Relative apical strain	0.91 ± 0.30
Base-to-apex gradient, mmHg	−12.69 ± 14.25
IVSd, mm	21.52 ± 4.30
LVMI, g/m^2^	198.69 ± 58.54
PWT, mm	18.20 ± 4.41
LVIDd, mm	33.95 ± 7.58
RWT	1.22 ± 0.67
E, m/s	0.96 ± 0.31
A, m/s	1.02 ± 0.39
E/A	1.07 ± 0.54
DT, ms	250.16 ± 96.28
E/e′	15.60 ± 5.63
LAVI, mL/m^2^	49.57 ± 16.67
MPG LVOT, mmHg	8.56 ± 12.57
PPG LVOT, mmHg	15.85 ± 22.65
V_max_ LVOT, m/s	1.75 ± 0.91
MPG AV, mmHg	41.19 ± 27.45
PPG AV, mmHg	75.72 ± 51.92
V_max_ AV, m/s	4.05 ± 1.62

EF: left ventricular ejection fraction; GLS: global longitudinal strain; IVSd: interventricular septal thickness; LVMI: left ventricular mass index; PWT: posterior wall thickness, LVIDd: left ventricular internal dimension in diastole, RWT: relative wall thickness; E: early diastolic filling velocity; A: late diastolic filling velocity; DT: deceleration time; LAVI: left atrial volume index; MPG LVOT: mean pressure gradient across the left ventricular outflow tract; PPG LVOT: peak pressure gradient across the left ventricular outflow tract; V_max_ LVOT: peak velocity across the left ventricular outflow tract; MPG AV: mean pressure gradient across the aortic valve; PPG AV: peak pressure gradient across the aortic valve; V_max_ AV: peak velocity across the aortic valve.

**Table 4:** Two-dimensional speckle-tracking analysis.

Parameters	
Mean basal strain, (%)	−12.30 ± 4.35
Mean mid-strain, (%)	−14.43 ± 3.29
Mean apical strain, (%)	−20.45 ± 5.41
Relative apical strain	0.91 ± 0.30
Base-to-apex gradient, mmHg	−12.69 ± 14.25

**Table 5:** Comparison between patients with histologically confirmed myocardial fibrosis and those without myocardial fibrosis.

	No MF (n = 7)	MF (n = 20)	*p*
Female n (%)	6 (85.7)	9 (45.0)	0.154
AVR n (%)	2 (28.6)	7 (35.0)	1.000
CABG n (%)	0 (0.0)	6 (30.0)	0.265
Age years	66.3 ± 11.2	66.4 ± 13.7	0.984
BSA m^2^	1.9 ± 0.2	1.9 ± 0.2	0.628
BMI kg/m^2^	32.1 ± 5.5	28.6 ± 5.4	0.151
Bleeding n (%)	0 (0.0)	4 (20.0)	0.507
Delirium n (%)	0 (0.0)	7 (35.0)	0.188
Pneumonia n (%)	1 (14.3)	7 (35.0)	0.581
Pacemaker n (%)	1 (14.3)	1 (5.0)	1.000
Stroke n (%)	0 (0.0)	2 (10.0)	0.975
Dialysis n (%)	0 (0.0)	2 (10.0)	0.975
Cardiogenic shock n (%)	0 (0.0)	2 (10.0)	0.975
CAD n (%)	1 (14.3)	11 (55.0)	0.154
AS n (%)	2 (28.6)	8 (40.0)	0.933
MI n (%)	6 (85.7)	8 (40.0)	0.100
AF n (%)	2 (28.6)	4 (20.0)	1.000
COPD n (%)	0 (0.0)	3 (15.0)	0.698
Diabetes mellitus n (%)	3 (42.9)	4 (20.0)	0.492
LOS days	12.4 ± 3.4	17.7 ± 11.5	0.246
ICU days	2.1 ± 0.9	7.9 ± 11.9	0.220
Wbc (10^9^/L)	10.5 ± 2.2	8.6 ± 3.4	0.184
Hb (g/dL)	11.5 ± 1.7	11.1 ± 2.0	0.606
Platelets (10^9^/L)	346.9 ± 103.8	302.7 ± 103.0	0.339
LDH U/L	366.1 ± 157.2	319.8 ± 110.8	0.401
Creatinine (mg/dL)	0.9 ± 0.2	1.0 ± 0.3	0.398
GFR (mL/min)	72.7 ± 18.8	73.7 ± 26.2	0.926
Potassium (mmol/L)	4.5 ± 0.2	4.3 ± 0.7	0.485
Arterial hypertension n (%)	6 (85.7)	18 (90.0)	1.000
Hyperlipidemia n (%)	2 (28.6)	3 (15.0)	0.818
NYHA >II	1 (14.3)	5 (25.0)	0.953
EF %	60.9 ± 8.3	65.2 ± 5.9	0.144
GLS %	−23.0 ± 5.7	−12.7 ± 2.7	**<0.001**
Mean basal-strain %	−17.3 ± 4.6	−10.6 ± 2.6	**<0.001**
Mean mid-strain %	−15.1 ± 4.0	−14.2 ± 3.1	0.548
Mean apical strain %	−16.7 ± 5.6	−21.8 ± 4.8	**0.032**
Relative apical strain	0.6 ± 0.1	1.0 ± 0.3	**0.003**
Base-to-apex gradient mmHg	−4.7 ± 5.5	−15.5 ± 15.4	0.085
IVSd mm	20.7 ± 3.4	21.8 ± 4.6	0.576
LVMI g/m²	165.5 ± 54.0	210.3 ± 56.8	0.081
PWT mm	17.6 ± 3.9	18.4 ± 4.7	0.668
LVIDd mm	32.3 ± 8.5	34.5 ± 7.4	0.510
RWT mm	1.3 ± 0.7	1.2 ± 0.7	0.656
E m/s	0.9 ± 0.3	1.0 ± 0.3	0.433
E/A	0.9 ± 0.2	1.1 ± 0.4	0.637
DT ms	247.6 ± 61.6	251.1 ± 107.2	0.905
E/e′	14.9 ± 5.4	15.8 ± 5.8	0.736
LAVI mL/m²	44.5 ± 10.0	51.4 ± 18.3	0.316
MPG LVOT mmHg	4.4 ± 1.2	10.0 ± 14.4	0.356
PPG LVOT mmHg	9.1 ± 2.5	18.2 ± 26.0	0.316
V_max_ LVOT m/s	1.5 ± 0.2	1.8 ± 1.0	0.368
MPG AV mmHg	21.9 ± 19.6	47.9 ± 26.9	0.387
PPG AV mmHg	39.5 ± 33.1	88.4 ± 51.9	**0.027**
V_max_ AV m/s	2.9 ± 1.3	4.5 ± 1.5	**0.029**
RVSD mmHg	27.0 ± 5.5	26.7 ± 6.1	0.944

For acronym definitions please refer to the legends of Table 2 and Table 3. Bold indicating statistically significance difference between the groups.

**Table 6:** Generalized linear logistic regression.

			95% Confidence Interval			
Names	SE	Odds Ratio	Lower	Upper	z	*p*
GLS %	0.01	1.07	1.05	1.09	6.42	**<0.001**
Mean apical strain %	0.01	1.00	0.98	1.03	0.09	0.929
Relative apical strain	0.24	1.30	0.81	2.08	1.10	0.285
Base-to-apex gradient	0.00	1.00	0.99	1.01	0.25	0.804
PPG AV mmHg	0.00	1.00	0.99	1.01	−0.71	0.488
V_max__AV m/s	0.15	1.09	0.81	1.45	0.56	0.585
Mean basal-strain %	0.02	1.00	0.97	1.03	−0.10	0.920

SE: standard error; for acronym definitions please refer to the legends of Table 2 and Table 3. Bold indicating statistically significance difference between the groups.

## Data Availability

All data are available through contact to the corresponding author.
